# Report of Special Committee on Diseases of the Eye

**Published:** 1864-07

**Authors:** E. L. Holmes

**Affiliations:** Chicago, Lecturer on Diseases of the Eye and Ear in Rush Medical College, and Surgeon of the Chicago Charitable Eye and Ear Infirmary


### CHICAGO MEDICAL JOURNAL.

VOL. XXI.] JULY, 1864. [No. 7.

### ORIGINAL COMMUNICATIONS.

### REPORT OF SPECIAL COMMITTEE ON DISEASES OF THE EYE.

By E. L HOLMES, M. D., of Chicago,

*Lecturer on Diseases of the Eye and Ear in Rush Medical College, and Surgeon of the Chicago Charitable Eye and Ear Infirmary.*

Presented to the Illinois State Medical Society, May, 1864.

Mr. President—Gentlemen:

Your committee would most respectfully report, that no contributions, either from members of this Society or from the Profession generally, have been received, although due notice of the appointment of this Committee was published in both medical journals of this city, with the request that the physicians of the State would aid the Committee in accomplishing the objects for which the Committee was appointed.

The following report, therefore, is composed entirely of materials collected in the personal experience of your Committee. In the first part of the report is a classification of the diseases, which have fallen under the observation of your Committee during the past eight years, and principally during the past five years.

The table may be supposed to present the relative number of cases of each disease, as found among patients with affections of the eye, in the North-west.

In the second part, your Committee had designed to furnish the history of several cases, which might be of particular interest to the profession; but thought best, without entering too much into detail, to ask the attention of the Society to some general principles, worthy of notice in the study of each class of disease.

Unfortunately, in several respects, the classification of diseases is imperfect. It is proper to state, that the numbers indicate number of patients and not of eyes affected.

The defect arose from the fact, that the annual reports of the Chicago Charitable Eye and Ear Infirmary, embracing more than 1400 patients, were prepared, not so much for scientific classification, as for the purpose of presenting to the public the number of patients treated, and the popular names of the diseases. When patients have been under treatment with each eye affected with a different disease, the most important alone was registered. And, whenever an eye was affected with several diseases, as, for instance, granular conjunctivitis, vascular cornea, trichiasis, entropion, or other complications, simply the primary disease has been recorded.

A few points in some sections of the following table, which may appear strange to members of the Society, will be subsequently explained:

| I DISEASES OF THE CONJUNCTIVA. | |
|---|---|
| Conjunctivitis Catarrhal, | 197 |
| do Granular, | 628 |
| do Purulent, | 32 |
| do Neonatorum, | 33 |
| do Pustular, | 114 |
| do Morbillous, | 21 |
| do Diphtheritic, | 3 |
| Injuries and Burns, | 67 |
| Xerophthalmia, | 4 |
| Pterygium, | 15 |
| Pinguicula, | 10 |
| Ecchymosis Spontaneous, | 4 |
| Total, | 1128 |

| II. DISEASES OF THE CORNEA. | |
|---|---|
| Corneitis Ulcerative, | 109 |
| do Superficial, | 19 |
| do Suppurative, Hypopion, | 7 |
| do do Onyx, | 5 |
| do do Abscess, | 11 |
| Staphyloma of Cornea, | 26 |
| Opacity of Cornea, | 64 |
| Tumor of do | 1 |
| Conical do | 2 |
| Motes on do | 61 |
| Total, | 305 |

III. DISEASES OF THE SCLEROTIC.

| | |
|---|---|
| Injuries of Sclerotic and Cornea, | 45 |
| Staphyloma of Sclerotic, | 2 |
| Episcleritis, | 2 |
| Total, | 49 |

IV. DISEASES OF THE LIDS.

| | |
|---|---|
| Ophthalmia Tarsi, | 34 |
| Trichiasis, | 40 |
| Entropion, | 19 |
| Ectropion, | 14 |
| Hypertrophy of Palpebral Integ., | 2 |
| Abscess of Lids, | 13 |
| Hordeolum, | 11 |
| Cystic Tumors, | 22 |
| Eczema, | 7 |
| Symblepheron, | 4 |
| Wounds, | 23 |
| Warts, | 1 |
| Ptosis, | 8 |
| Lupus, | 1 |
| Oedema, | 15 |
| Nevus, | 2 |
| Molluscum of Lid, | 1 |
| Total, | 217 |

V. DISEASES OF THE IRIS.

| | |
|---|---|
| Iritis, | 52 |
| do Syphilitic, | 13 |
| Iridochoroiditis, | 13 |
| Occlusion of Pupil, | 15 |
| Coloboma of Iris and Choroid, | 4 |
| Mydriasis, | 3 |
| Wounds of Iris, | 3 |
| Adhesion of Iris and Lens, | 10 |
| Total, | 113 |

VI. DISEASES OF THE CHOROID, ETC.

| | |
|---|---|
| Choroiditis, | 21 |
| do Pigmentosa, | 22 |
| Sclero Choroiditis, | 16 |
| Staphyloma Posticum, | 4 |
| Opacities of Vitreous Humor, | 31 |
| Coagulum in Vitreous Humor, | 1 |
| Glaucoma, | 3 |
| Total, | 98 |

VII. DISEASES OF THE RETINA.

| | |
|---|---|
| Retinitis, | 20 |
| do of Bright's Disease, | 4 |
| Congestion of Retina, | 17 |
| Detachment of Retina, | 5 |
| Atrophy of Optic Nerve, (papilla,) | 3 |
| Hemeralopia, | 2 |
| Embolie of Art Cintralis, | 1 |
| Cancer of Retina, | 5 |
| Muscæ Volitantes, | 9 |
| Extravasation of blood under retina, | 2 |
| Amblyopia, | 32 |
| Amaurosis, | 35 |
| Total, | 135 |

VIII. DISEASES OF THE LENS.

| | |
|---|---|
| Cataract Incipiens, | 13 |
| do Hard, | 20 |
| do Soft, | 17 |
| do Pyramidal, | 11 |
| do Congenital, | 2 |
| do Trumatic, | 12 |
| do do layer, | 6 |
| Dislocation of Lens Traumatic, | 2 |
| do do Spontaneous, | 1 |
| Total, | 84 |

IX. DISEASES OF THE GLOBE.

| | |
|---|---|
| Atrophy of Globe, | 14 |
| Fungus Hæmatodes, | 1 |
| Hydrophthralmos, | 2 |
| Microphthalmos, | 2 |
| Sympathetic Ophthalmitis, | 8 |
| Total, | 27 |

X. DISEASES OF THE MUSCLES.

| | |
|---|---|
| Strabismus, | 19 |
| Paralysis of 3d Pair of Nerves, | 7 |
| do 4th do do | 4 |
| do 6th do do | 1 |
| Blepherospasm, | 2 |
| Nystagmus, | 12 |
| Total, | 45 |

XI. DISEASES OF THE LACHRYMAL APPENDAGES.

| | |
|---|---|
| Abscess of Lachrymal Sack, | 5 |
| Obstruction of Nasal Duct, | 20 |
| Congenital Fistula of Sack, | 1 |
| Obliteration of Canalliculi, | 1 |
| Passage of air through do | 2 |
| Eversion of Punctum Lachrymal, | 3 |
| Total, | 32 |

| XII. DISEASES OF THE ACCOMMODATIVE APPARATUS. | |
|---|---|
| Myopia, | 8 |
| Presbyopia, | 3 |
| Asthenopœa, | 35 |
| Hypermetropia, | 2 |
| Total, | 48 |

| XIII. DISEASES OF THE ORBIT. | |
|---|---|
| Necrosis of Orbit, | 1 |
| Carcenoma of Orbit, | 1 |
| Aneurism of Orbit, | 1 |
| Neuralgia, | 5 |
| Total, | 8 |
| Total of all Diseases of the Eye, | 2289 |

From this table it will be observed, that diseases of the conjunctiva form by far a greater class, than those of any other portion of the eye. To this class may be added a large part of the diseases of the lids and cornea, since they are but sequelæ of conjunctivitis. A large number of blind patients, that have been examined, lost their vision as a result of this disease, and there are reasons for believing that a very large portion of the blind in the blind asylum, and other portions of the State, lost their sight from neglected or maltreated inflammation of the conjunctiva.

In many parts of Illinois and the North-west, these diseases are very common, and are the cause of more pain and misery than almost any other disease. Your Committee, therefore, believes it is the duty of every practitioner in the Western States to make conjunctivitis the subject of special study. Every opportunity should be provided medical students for the clinical study of these diseases and their treatment.

A few words regarding the causes of conjunctivitis at the West, are perhaps not out of place, though little can be said in addition to what has already been reported at previous meetings of this Society. The opinions, formerly expressed, were almost entirely founded upon the views and experiences of others, since there has not been, during the past eight years, a single epidemic of conjunctivitis in Chicago. An honored member of this Association, who has enjoyed a long experience and extensive opportunities of observation, has informed your Committee, that he believes epidemics of conjunctivitis are not dependent upon the prevalence of dust or upon the dryness of the atmosphere, since they have often occurred when the prairies were covered with moisture. It is true, few places experience more violent winds, loaded with more dust, than Chicago; and yet, as has been stated, epidemic conjunctivitis is perhaps unknown in this city. Still, physicians generally seem to consider these agents as among the most active causes of the disease.

It is certainly to be hoped that all members of this Society, who may have an opportunity of observing epidemics of conjunctival inflammation, will report all facts relating to the condition of the atmosphere, surface of the country, the habits and occupation of the patients.

One word only in regard to treatment. In ordinary uncomplicated cases of acute conjunctivitis, your Committee is convinced that success in treatment depends principally upon the skillful use of caustic astringents. They should not be dropped into the eye, but applied directly to the mucous membrane of the lids, by means of a delicate brush, the secretions having first been removed by gentle applications of a bit of linen.

There is reason to believe, in the treatment of chronic conjunctivitis, that practitioners, generally, in the West, use these remedies too strong. Slight applications of the crystal sulphate of copper have given best satisfaction to your Committee in the largest proportion of cases.

Four typical cases of Xerophthalmia have been observed. One was remarkable, as resulting from phlyctenular conjunctivitis. The patient was five years of age; the palpebral conjunctiva of the right eye was totally absorbed, and the edges of the lids brought in direct contact with the cornea, which was covered with a dry translucent membrane like parchment.

But three cases of true diphtheritic conjunctivitis have been noticed. Unfortunately two cases in children terminated with almost total destruction of vision; your Committee was discharged from the third case in consequence of the unfavorable prognosis which was given. The subsequent history of the case is unknown. There is reason to believe that this disease, not uncommon in Europe, is quite rare in this country, although the corresponding affection of the throat is at times fearfully prevalent.

Nearly one-seventh of all the cases above enumerated are affections of the cornea. Many interesting points connected with these diseases, especially in children, are worthy of separate papers. Although quite a large number of cases of corneitis in children were accompanied with caries of the teeth, only four examples have been observed in which the teeth have been notched, as described by Mr. Hutchinson. By far the greater part of the patients affected with primary corneal disease have been of an unhealthy diathesis; a few cases only being in patients in whom the health was apparently perfect.

The treatment of certain injuries of the cornea and sclerotic will be discussed in connection with sympathetic ophthalmitis.

There have been but few cases of diseases of the lids, which have presented points worthy of remark in a report like this. One case of molluscum of the upper lid is mentioned as the only one ever observed by your Committee in his own practice or in that of others.

In the last report of this Committee, your attention was called to the treatment of certain forms chronic conjunctivitis, recommended in the first volume of Archives for Ophthalmology, and in the annual report of Pagenstecher and Saemisch. This treatment, which consists in elongating the palpebral fissure and in introducing vertical stitches throught the integument and the orbicular muscle of the upper lid, has been found of special service in spasm of the lids, with tendency to entropion, and union of the lids at the external angle.

Certain cases of trichiasis, attended with great atrophy of the palpebral conjunctiva and of the tarsal cartilage itself, have been found difficult to relieve. The bulbs of the cilia appear so deep and misplaced in the tissues, that any efforts to remove them by scalping the edge of the lids, only add to the atrophy and fail to improve the condition of the organ. Fortunately, however, such cases are not frequent.

About five per cent. of the patients treated have been with affections of the iris. Your Committee has never attempted to conduct the treatment of iritis without the use of mercury, although high authority has shown that the disease may thus be successfully treated. Success with the use of calomel and large and repeated doses of atropia locally, have created an unwillingness to modify this plan of treatment. Excision of a part of the iris, as recommended by Graefe, in chronic iritis with attachments of the iris to the lens, has been performed apparently with great benefit in three cases.

Three cases of injuries of the iris are somewhat remarkable. Two are cases of detachment of the iris from the ciliary muscle, produced by slight blows upon the eye. The separation extended about one-eighth of the circumference, in one case in the upper and outer, and in the other in the upper and inner quarter of the iris. In the former, recovery with almost perfect vision, but with a double pupil, was the result. In the latter, there was a second pupil and a traumatic cataract. The other case of injury was even more remarkable. A boy, seven years of age, received the sharp point of a pair of scissors in the right eye, which penetrated the cornea, iris, and possibly the lens, near the middle of the lower and outer quarter of these organs. The wound in the cornea healed with a very faint nebulous cicatrix; the iris was left with a small but permanent opening at the point of injury. No opacity of the lens could be perceived. Vision remained perfect. The treatment was simply low diet, absolute rest, and wet compresses.

The classification of the diseases of the vitreous humor, choroid and retina has been based entirely upon the abnormal changes, discovered by means of the ophthalmoscope. Whenever the line of demarkation between the papilla of the optic nerve and the retina has been indistinct, presenting the appearance represented by tables X, figure 4, of Liebreich's Plates, and tables X and XI of Jaeger's, the disease has been classified as retinitis. In those cases where absorption and deposition of pigment have been observed, with or without the peculiar yellow-colored patches so often seen, the disease has been termed choroiditis. Ophtalmologists are apparently not all satisfied with the term retinitis pigmentosa. There is reason to believe in many cases the disease is an affection of the choroid as well as of the retina. In no instance has your Committee found this disease connected with consanguinity or idiocy, as observed by some writers.

Four cases of characteristic disease of the retina in patients affected with morbus Brightii, have been carefully studied. It is a matter of interest to ascertain the proportion of patients with this disease of the kidneys, who also suffer from amaurotic symptoms. A large number of patients with amaurotic symptoms have applied for treatment, where an examination with the ophthaloscope was not permitted. These diseases were simply recorded as amblyopia or amaurosis, and no treatment instituted.

Glawcoma is evidently not a common disease at the West. Only three cases of this affection, and no others with dilated pupil and abnormal hardness of the globe, has been observed in Chicago, by your Committee.

As your Committee intends, at some future time, to make cataract the subject of a special report, nothing need be said at present upon the cases in the section of the above table devoted to diseases of the lens.

Eight cases of sympathetic ophthalmitis have been observed with total loss of sight, in which perfect vision of one eye could evidently have been saved by the early removal of the eye primarily injured. Quite a large number of cases have been noticed, where punctured and incised wounds of the globe have been followed by long distressing inflammation of the eye. No treatment seemed to afford relief till, after weeks and months of suffering, the patients permitted the extirpation of the eye. It is true the majority of such injuries heal without these violent symptoms, and your Committee would not urge an indiscriminate mutilation of these patients. But is it not a question worthy of consideration, as stated in the last report, whether it is not better to sacrifice a sightless and inflamed eye after due delay, than to endeavor to save the form of the eye simply, at the risk of total blindness? It is necessary to distinguish one very common form of sympathy in these cases, from true sympathetic ophthalmitis. The first is simply a mild degree of irritation, with secretion of tears, and slight photophobia in one eye, when the other is excited, as for example, by the presence of a minute object under the lid. The other is a dangerous inflammation of the choroid, iris and retina, which, if it has once commenced, there is reason to believe, is seldom relieved by the removal of the other eye. Hence your Committee would urge upon the general practitioner the propriety of operating before this serious form of disease has supervened.

Although the abscision of the cornea and iris is often attended with good results, the experience of many of the most celebrated oculists seems to favor the total extirpation of the eye.

One case of congenital fistula of the sack, in a boy seven years age, and two cases in which considerable annoyance was caused by the passage of air through the canallicnli on blowing the nose, are simply worthy of attention as being unusual. The operation of slitting the canalliculi, as recommended by Bowman in cases of eversion of the puncta, and the use of injections in the early stages of obstructions of the nasal duct are among the most satisfactory operations in ophthalmic surgery. Your Committee, although unwilling for slight causes, to sacrifice an organ, yet believes, in view of the want of pa tience and fortitude on the part of so many, especially the poor, suffering from chronic and neglected diseases of the duct, that much discomfort can be prevented by the obliteration of the sack.

No cases of more than ordinary interest, in diseases of the muscles have been noted.

But few cases of anomalies of the refracting media have been under the care of your Committee. This is explained by the fact that patients suffering from myopia and presbyopia usually apply to the optician, rather than the oculist, for advice. Your Committee has not been in the habit of making careful examinations of eyes affected with strabismus, in reference to the existence of myopia or hypermetropia, neither has he instituted suitable investigations to determine whether asthenopia was dedendent upon weakness of the recti muscles, or upon hypermetropia—formerly termed excessive presbyopia.

Not a single case of astygmatism, or difference of convexity or density of the refracting media in different meridians, has been detected, although no investigations were made by your Committee till within two years.

The attention of the Society is called to the experiments upon the properties of the Calabar bean as recently described in nearly all the leading journals of medicine.

A few remarks upon the status of ophthalmic literature in America, may not be inappropriate. Comparatively few works of merit, on diseases of the eye, have been writter in this country. Nearly all our books are simply reprints of English works, which are now in many respects much behind the advance of science. Probably it is not too much to affirm that in the English language, there does not exist a complete and desirable text-book on diseases of the eye. It is true, the works of Mackenzie, Lawrence and others, embracing valuable monographs, furnish the student with a vast amount of practical knowledge of many ophthalmic diseases. But they are not what the profession now demands—a complete work, corresponding, for instance, to that of Stellwag, of Vienna, with a systematic arrangement of the diseases of each tissue, with its anatomy and physiology carefully and clearly discussed, as also the pathology of each abnormal process, followed by a description of the most approved treatment. There are in our country those who, with their own extensive experience, and with their study of the works of Graefe, Donders, Wells, Jaeger, in fact, of all eminent writers in every language, are able to contribute such a work to our literature. It will require immense labor, but it is to be hoped some one will soon commence it.

The *American Journal of Ophthalmology*, edited by Dr.

Homberger, merits your support, and will most amply repay members of our profession for its perusal.

The attention of the Society is this year again called to the condition of the Chicago Charitable Eye and Ear Infirmary. This instutution has now entered upon the seventh year of its existence, during which, 1682 patients have been treated; 1272 with diseases of the eye, and 410 with diseases of the ear. The association consists of a board of twelve trustees, and a Board of two consulting and two attending surgeons. The operations of the Infirmary have thus far been much limited from want of means, which have been sufficient, merely to furnish poor patients with treatment at its Dispensary. The good which has even thus been accomplished, is almost incalculable. Not unfrequently, however, patients have suffered from want of suitable diet and care, and of protection from exposure. It must certainly be a matter of interest to this Society, and to the profession generally, to learn that the Infirmary has been placed in a position in which its usefulness, as is hoped, will be widely extended.

The President of the Board of Trustees, Walter L. Newberry, Esq., has donated the lease of a valuable lot of land to the Infirmary for the term of ten years. A good and commodious building has already been secured for a hospital, and efforts are now being made by private subscriptions to furnish it in such a manner as will be comfortable to patients and creditable to the city. It must be understood that this charity, at present, consists in providing the poor with comfortable apartments and treatment for diseases of the eye or ear, gratuitously. A small sum per week, hereafter to be determined, will be required for board, since the funds of the Infirmary are not sufficient to furnish this last to patients gratuitously. Efforts will be made in due time to secure a fund for this purpose also. Will not the profession lend this Institution the support and encouragement it merits?

At the last meeting of the Board of Trustees the following resolution was passed: That students of medicine be admitted to the Infirmary, with the privilege of studying diseases of the eye and ear, under such rules as the surgeons may, from time to time, deem best.

### RAMBLES IN MILITARY HOSPITALS.

REPORT OF

